# Specific DNA identification of Pheretima in the Naoxintong capsule

**DOI:** 10.1186/s13020-019-0264-7

**Published:** 2019-09-30

**Authors:** Xiaoxiao Zhu, Hoi-Yan Wu, Pang-Chui Shaw, Wei Peng, Weiwei Su

**Affiliations:** 1 Guangdong Engineering and Technology Research Center for Quality and Efficacy Reevaluation of Post-Market Traditional Chinese Medicine, Guangdong Key Laboratory of Plant Resources, School of Life Sciences, Sun Yat-Sen University, Guangzhou, 510275 China; 2 Li Dak Sum Yip Yio Chin R & D Centre for Chinese Medicine, The Chinese University of Hong Kong, Shatin, N.T., Hong Kong China; 3 Institute of Chinese Medicine, The Chinese University of Hong Kong, Shatin, N.T., Hong Kong China; 4 School of Life Sciences, The Chinese University of Hong Kong, Shatin, N.T., Hong Kong China

**Keywords:** Pheretima, Naoxintong capsule, DNA identification, COI gene

## Abstract

**Background:**

Pheretima is a minister drug in Naoxintong capsule (NXTC), a well-known traditional Chinese medicine (TCM) formula for the treatment of cardiovascular and cerebrovascular diseases. Owing to the loss of morphological and microscopic characteristics and the lack of recognized chemical marker, it is difficult to identify Pheretima in NXTC. This study aims to evaluate the feasibility of using DNA techniques to authenticate Pheretima, especially when it is processed into NXTC.

**Methods:**

DNA was extracted from crude drugs of the genuine and adulterant species, as well as nine batches of NXTCs. Based on mitochondrial cytochrome *c* oxidase subunit I (COI) gene, specific primers were designed for two genera of genuine species, *Metaphire* and *Amynthas*, respectively. PCR amplification was performed with the designed primers on crude drugs of Pheretima and NXTCs. The purified PCR products were sequenced and the obtained sequences were identified to species level with top hit of similarity with BLAST against GenBank nucleotide database.

**Results:**

Primers MF2R2 and AF3R1 could amplify specific DNA fragments with sizes around 230–250 bp, both in crude drugs and NXTC. With sequencing and the BLAST search, identities of the tested samples were found.

**Conclusion:**

This study indicated that the molecular approach is effective for identifying Pheretima in NXTC. Therefore, DNA identification may contribute to the quality control and assurance of NXTC.

## Background

Naoxintong capsule (NXTC), a traditional Chinese medicine (TCM) for the prevention and treatment of cardiovascular and cerebrovascular diseases, is approved by the China Food and Drug Administration (CFDA) and recorded in the 2015 edition of Chinese Pharmacopoeia (ChP 2015) [1]. NXTC is developed from Bu-Yang-Huan-Wu-Tang, a traditional prescription which has been found effective clinically in the treatment of stroke and cerebral infarction since Qing dynasty [2]. Nowadays, several comprehensive and systematic studies have confirmed the clinical efficacy of NXTC for the treatment of patients with angina pectoris [3], cerebral infarction [4], transient ischemic attack [5], vertebra-basilar insufficiency [6] and carotid atherosclerosis [7, 8].

Each NXTC weighs 0.4 g and is composed of sixteen powdered TCMs, including thirteen plant drugs and three animal drugs, namely Pheretima, Hirudo and Scorpio. These three animal drugs account for only 16.7% (w/w) of all sixteen TCMs, but they are the major effective components of NXTC. They play vital roles in clearing and activating the meridians and collaterals [2]. Pheretima was documented as a TCM in the earliest known treatise of Chinese material medica, “Shennong Bencao Jing”, written 2000 years ago [9], and was widely used for the treatment of fever, asthma and stroke [1]. Increasing evidences indicate that Pheretima has wide therapeutic properties, including anti-inflammatory activity [10], anti-oxidative activity [11], fibrinolytic activity [12], anti-asthmatic activity [13], as well as promoting tumor apoptosis [14] and bone regeneration [15]. According to ChP 2015, there are four Pheretima animals, namely *Amynthas aspergillus* E. Perrier, *Amynthas pectiniferus* Michaelsen, *Metaphire vulgaris* Chen and *Metaphire guillelmi* Michaelsen. However, according to a survey on the resources of Pheretima and examination of the available drug [16], there are thirteen original animals of Pheretima in the market, including some common adulterants not recorded in ChP 2015. In consideration of the efficacy and safety of Pheretima, it is necessary to authenticate the species accurately. Unlike plant drugs, animal drugs lack the representative markers or chemical profiles. In ChP 2015, morphological and microscopic observation and thin layer chromatography (TLC) are used to identify Pheretima. However, the above conventional methods lack specificity and depend on experience of the examiner. What is more, the morphological and microscopic characteristics of the constituting species are lost in NXTC due to the processing procedure. Thus, it is difficult to find whether the NXTC contains the genuine species of Pheretima.

DNA is unique to every individual and exists in most biological tissues. In recent years, DNA techniques have been widely applied in the identification of TCM [17, 18], and it has also been used to identify Pheretima [19–21]. In addition, DNA methods have been established to authenticate two medicinal snakes (Zaocys and Agkistrodon) and one Chinese herb (Fritillariae cirrhosae bulbus) in ChP 2015. Nevertheless, previous studies mainly focused on single TCM rather than complex prescriptions. In this study, we explored the possibility of using DNA techniques to identify Pheretima in NXTC.

## Methods

### Sample collection

Pheretima medicinal materials were collected from various pharmacies in China or provided by Shaanxi Buchang Pharmaceuticals Co., Ltd. (Shaanxi, China) (Table 1). All of them were authenticated based on their morphological features by Guangdong Institute for Drug Control (Guangdong, China) according to ChP 2015 and confirmed by DNA sequencing using the universal primers of mitochondrial cytochrome *c* oxidase subunit I (COI) [22] (primer sequences are shown in Table 2). These authenticated Pheretima samples were deposited in School of Life Sciences, Sun Yat-Sen University. Nine batches of NXTC were provided by Shaanxi Buchang Pharmaceuticals Co., Ltd. (Shaanxi, China). In-house NXTCs were also prepared as controls according to the standardized procedure for the preparation of NXTC in ChP 2015. A total of seven batches of homemade NXTC using different identified genuine or adulterant Pheretima samples and one batch of Pheretima-deficient NXTC containing all listed TCMs except for Pheretima were made. Detailed information of the samples and materials are shown in Table 1.

**Table 1:** Information of the studied samples

Material	Description	Batch no.	Source	Sample identity
Pheretima	Genuine	P1–P3	Buchang	*Metaphire vulgaris*
		P4	Buchang	*Metaphire guillelmi*
		P5	Market	*Metaphire vulgaris*
		P6–P10	Market	*Amynthas aspergillus*
	Adulterant	P11	Market	*Metaphire magna* ssp.
		P12	Market	N/A
		P13	Market	N/A
		P14	Market	N/A
		P15	Market	*Metaphire californica*
NXT capsules		N1–N9	Buchang	Pheretima: *Metaphire vulgaris*
		N10	Homemade	Pheretima: P6
		N11	Homemade	Pheretima: P4
		N13	Homemade	Pheretima: P11
		N14	Homemade	Pheretima: P12
		N15	Homemade	Pheretima: P13
		N16	Homemade	Pheretima: P14
		N17	Homemade	Pheretima: P15
Pheretima-deficient NXTC		T4840	Homemade	

* All the crude drugs were identified via their morphological features by Guangdong Institute for Drug Control (Guangdong, China) according to ChP 2015 and confirmed by DNA sequencing using the universal primers of COI. Buchang: Shaanxi Buchang Pharmaceuticals Co., Ltd. (Shaanxi, China)

*N/A* not available

**Table 2:** Primers for PCR amplification and sequencing

Primer name	Sequence (5′ → 3′)	Amplicon size (bp)	Annealing temperature (°C)
Universal primers of COI [22]			
LCO1490	GGTCAACAAATCATAAAGATATTGG	650	40
HCO2198	TAAACTTTCAGGGTGACCAAAAAATCA		
MF2R2			
COI Metaphire F2	TTAGTGTCGTCCGCCGCAGTT	232	58
COI Metaphire R2	CTACTGCCCACACAAATAGTGGG		
AF3R1			
COI AA F3	TTTGGAAACTGACTGCTCCCA	247	55
COI AA R1	CTAAAATTGATGAGGCACCC		

### DNA extraction

DNA was extracted from crude drugs with Rapid Genomic DNA kit (Biomed, Beijing, China). Broad-spectrum plant Rapid Genomic DNA kit (Biomed, Beijing, China) was used for NXTC according to manufacturers’ instructions with brief modification, respectively. The kit for plant DNA extraction was chosen as 83.6% (w/w) of NXTC is made up of plant materials. Twenty microliter proteinase K (20 mg/ml, Biomed, China) was added at the first step to digest the animal drugs. The DNA of Pheretima-deficient NXTC and seven batches of homemade NXTC were extracted by the same means as NXTC. Finally, 100 μl DNA solution was obtained and the concentration was measured by NanoDrop 2000C spectrophotometer (Thermo Fisher Scientific, US). DNA was then adjusted to 50.00 ng/μl.

### Primer design

DNA sequences of COI gene for species belonging to *Metaphire* genus and *Amynthas* genus (Additional file 1: Tables S1-1 to S1-2) were obtained from GenBank of National Center for Biotechnology Information (NCBI) and aligned using BioEdit 7 software [23]. Owing to the significant difference of DNA sequences between *Metaphire* genus and *Amynthas* genus, different primers were designed respectively, according to the polymorphic sites between the genuine and adulterant species. All the primers were designed using Primer 3 (http://primer3.ut.ee/) and the information is shown in Table 2.

### PCR amplification and DNA sequencing

PCR was performed in a 15 μl reaction mixture containing 1.5 μl 10× PCR buffer (200 mM Tris–HCl, pH 8.4, 200 mM KCl, 100 mM (NH4)_2_SO_4_), 1.5 μl 25 mM MgCl_2_, 1.2 μl 2.5 mM dNTP mixture, 0.75 μl 10 μM each of the forward and reverse primer, 0.5 μl DNA sample and 0.1 μl 5 U/μl *Taq* polymerase. All the buffers and chemicals mentioned above were bought from Beijing Biomed Co., Ltd (Beijing, China). Reactions were conducted using Veriti 96-well Thermal Cycle (Applied Biosystems, Singapore) through initial denaturation at 95 °C for 3 min, then 39 cycles of denaturation at 95 °C for 45 s, annealing at indicated temperature for 45 s and extension at 72 °C for 1 min, with a final extension at 72 °C for 5 min. The PCR products were electrophoresed and visualized on 1.5% agarose gels stained with SYBR Safe DNA gel stain (Thermo Fisher Scientific, US), purified with DNA gel purification kit (Biomed, Beijing, China) and sequenced by Sanger sequencing (Tech Dragon, Hong Kong and Beijing Genomics Institute, Guangzhou, China). By means of Basic Local Alignment Search Tool (BLAST) of NCBI, the amplified sequences were compared with public DNA sequences in GenBank nucleotide database to match the most likely species with the top hit.

## Results

### Identification of the animal drug by species-specific PCR

**Fig. 1:**
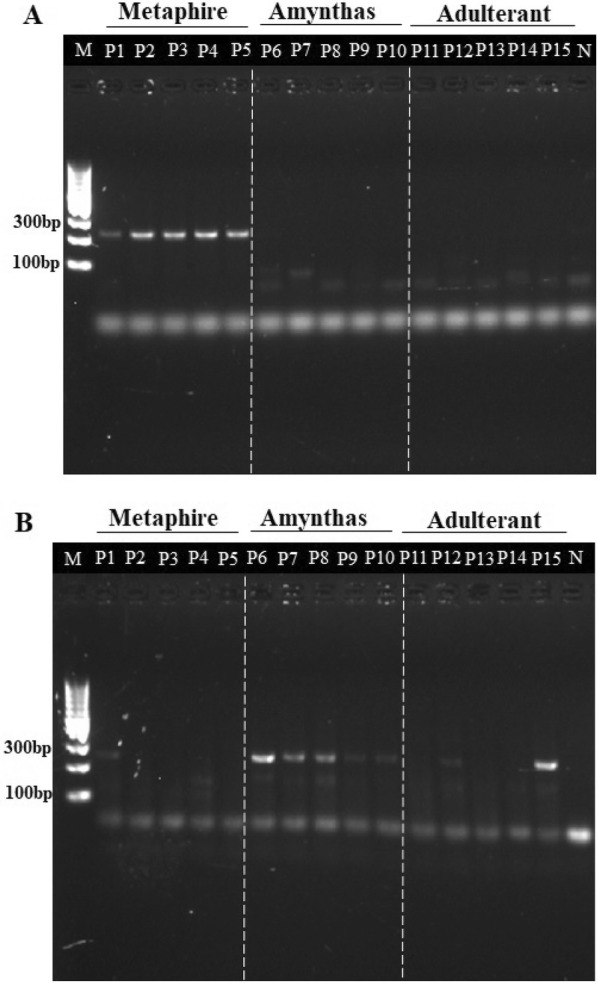
Specific primers for amplifying DNA extracted from the crude drugs of Pheretima. DNA was amplified using specific primers, (**a**) primers MF2R2 for *Metaphire* genus and (**b**) primers AF3R1 for *Amynthas* genus, respectively. Adulterant numbered P15 could also be amplified using primers AF3R1, but with sequence and BLAST to the adulterant. PCR without DNA template was used as negative control (N). M:DNA ladders

To verify the specificity of the designed primers, crude drugs for both the genuine and the adulterant were firstly tested. The common adulterants of Pheretima we collected on the market include *M. magna* ssp. and *M. californica* (Kinberg), as well as another three unidentifiable species. As shown in Fig. 1a, by using the specific primers MF2R2 for *Metaphire* genus, 232-bp fragments were amplified in the samples from P1 to P5. At the same time, no amplification was obtained from another genuine species in the *Amynthas* genus (P6-P10) and the adulterants (P11-P15). For AF3R1(Fig. 1b), the specific primers for *Amynthas* genus, 247-bp amplicons with sequences the same as the genuine species (*A. aspergillus*) were obtained, while no amplification was obtained from samples of *Metaphire* genus and most of the adulterants. One of the adulterants, sample P15, was also amplified by AF3R1 and found as *M. californica* by DNA sequencing. It was identified to be *M. californica* by using the universal primers of COI. To sum up, as for the Pheretima, primers MF2R2 could distinguish *M. vulgaris* and *M. guillelmi* from the *Amynthas* genus and the adulterants. Whereas, though primers AF3R1 could differentiate *A. aspergillus* from the *Metaphire* genus, sequencing and BLAST need to be done to confirm the amplicon is the genuine species or the adulterant (Additional file 2: Fig. S2-2).

Among all the medicinal materials tested above, P1–P4 were provided by Shaanxi Buchang Pharmaceuticals Co., Ltd. (Shaanxi, China) and were identified as *M. vulgaris* or *M. guillelmi* (Table 1). On this occasion, the results shown above indicated that the primers MF2R2 could be used to identify the genuine species of Pheretima used, which would contribute to the quality control of NXTC at product manufacturing.

### Identification of the animal drugs in NXTC by species-specific PCR

Based on the successful identification of crude drugs, all the specific primers were tested in authenticating the three animal drugs in nine batches of NXTC. It should be noticed that since the company purchased the crude drugs from fixed suppliers, in principle only *M. vulgaris* and *M. guillelmi* would be detected in NXTC.

**Fig. 2:**
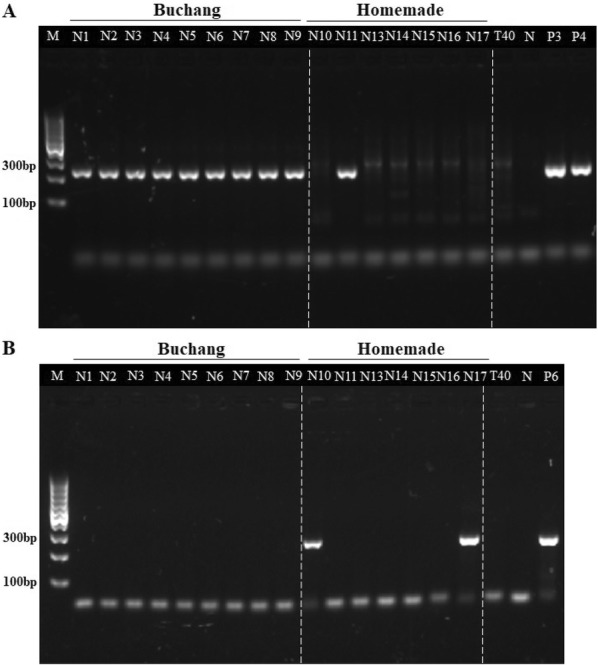
Species-specific primers for amplifying Pheretima in NXTC. In the nine batches of NXTCfrom the company, DNA was amplified using (**a**) primers MF2R2, the species-specific primers for Metaphire genus. For (**b**) primers AF3R1, the species-specific primers for Amynthas genus, no amplification was obtained. Seven batches of homemade-NXTCs were made, respectively with the identified crude drugs of Pheretima numbered P6, P4, P11, P12, P13, P14, P15 for each batch. Pheretima-deficient NXTC (T40) was made as the negative control and the identified samples of genuine species (P3: *M. vulgaris*, P4: *M. guillelmi* and P6: *A. aspergillus*) were used as positive control. M:DNA ladders, N:PCR without DNA template

As shown in Fig. 2b, no amplification was obtained in NXTC while using primers AF3R1, indicating that there is no *A. aspergillus* in NXTC. However, 232-bp fragments were amplified in NXTC using primer MF2R2 (Fig. 2a) and all the amplicons were sequenced and BLAST to *M. vulgaris*, which is one of the two species the company used (Additional file 3). To find whether primers MF2R2 and AF3R1 could authenticate Pheretima in other NXTC, we tested seven batches of homemade NXTC, in which *A. aspergillus*, *M. guillelmi* and the other five batches of adulterants were included respectively. With primers MF2R2, *M. guillelmi* could be identified when it was included in sample N11 and the NXTC including the adulterants could also be differentiated. At the same time, *A. aspergillus* and the other adulterants could be distinguished when they were included in NXTC. As revealed above, the Pheretima that the company used include two species, but only *M. vulgaris* was detected in these nine batches of NXTC, which probably due to the use of *M. vulgaris* more frequently. Accordingly, with primer MF2R2, it could be confirmed whether the NXTC to be tested contains the genuine species of Pheretima.

### The sensitivity test of NXTC using specific primers for Pheretima

**Fig. 3:**
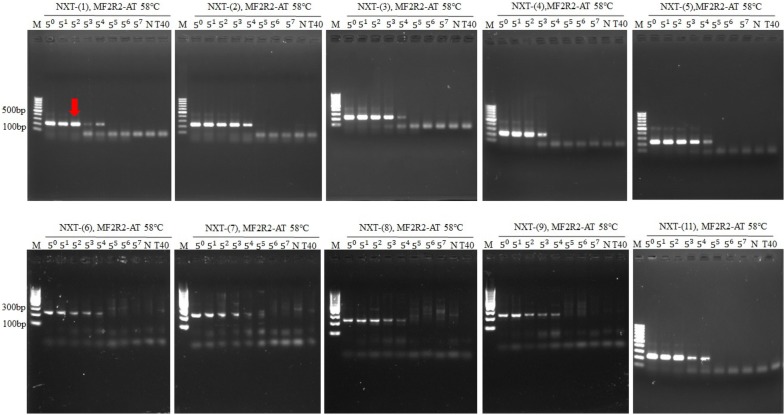
The sensitivity test of NXTC using primers MF2R2. DNA templates of NXTC (50 ng/μl) (5^0^) were fivefold serial-diluted (5^1^ to 5^7^) and were amplified using specific primers MF2R2. The suggested detection limit of DNA concentration of NXTC was 2 ng/μl, which was indicated by red arrow. Pheretima-deficient NXTC (T40) was made as the negative control. M:DNA ladders, N:PCR without DNA template

**Fig. 4:**
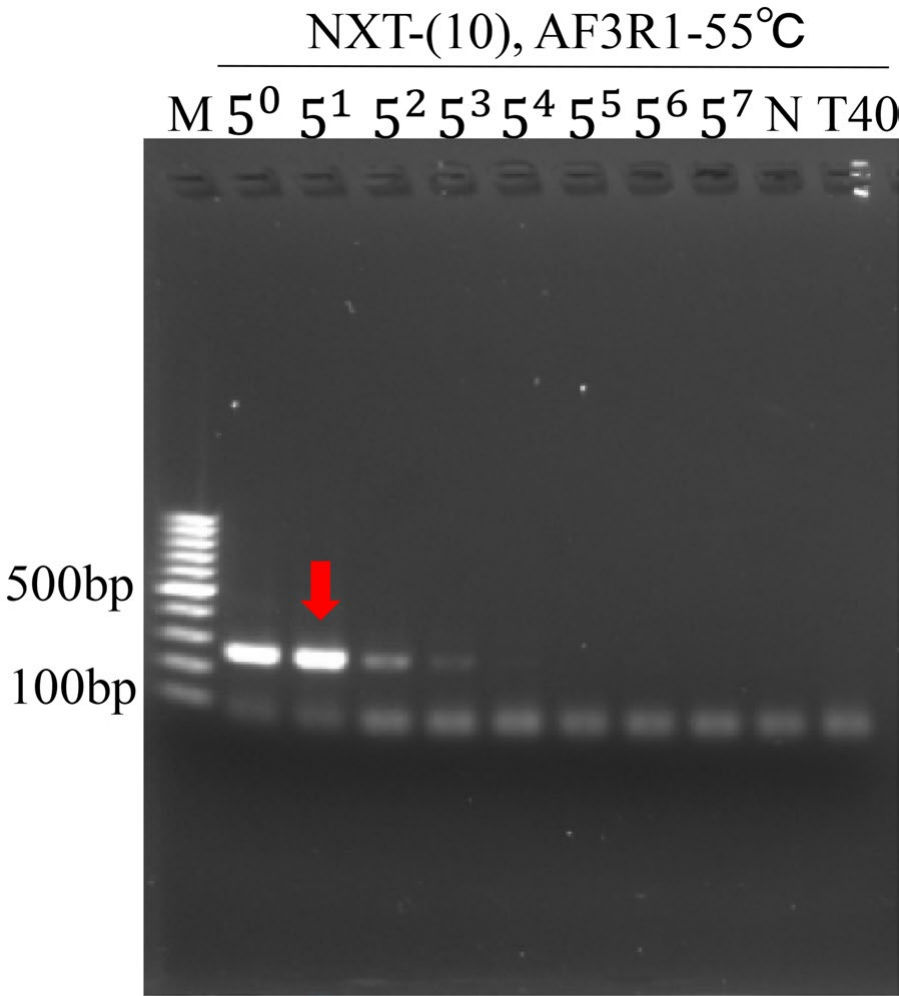
The sensitivity test of NXTC using primers AF3R1. DNA templates of NXTC (50 ng/μl) (5^0^) were fivefold serial-diluted (5^1^ to 5^7^) and were amplified using specific primers AF3R1. The suggested detection limit of DNA concentration of NXTC was 10 ng/μl, which was indicated by red arrow. Pheretima-deficient NXTC (T40) was made as the negative control. M:DNA ladders, N:PCR without DNA template

DNA templates of NXTC (50 ng/μl) (5^0^) were fivefold serial-diluted (5^1^ to 5^7^) and were amplified using specific primers for Pheretima. Every batch of concerned NXTC was tested and repeated once. Results showed that the animal drugs’ DNA was detectable with the DNA concentration of NXTC around 50 ng/μl for all the specific primers, and even with lower concentration (Figs. 3 and 4). Taken all the concerned NXTC into consideration, we suggest that the DNA concentration of tested NXTC should be at least 2 ng/μl with primers MF2R2 or 10 ng/μl with primers AF3R1 if this method was applied in the quality control of NXTC.

## Discussion

Accurate species identification is crucial to the authenticity, safety and efficacy of medicinal products. In addition to the conventional methods, such as organoleptic and chemical methods, species identification of animal-derived products mainly relies on protein [24] and DNA [25, 26] analysis. Protein analysis is usually suitable for raw materials. But for animal-based TCMs, their proteins are generally destroyed due to the high temperature or high salinity during the process. DNA is more reliable and stable than other macromolecules and is found in all tissues. In ChP 2015, “Principles for molecular identification of traditional Chinese materia medica using DNA barcoding” was established to guide the molecular identification of TCM [27]. DNA barcoding is the use of a short, conserved and standardized sequence of the genome to identify species [22]. The application of DNA barcoding for the species identification of global species has become routine. DNA authentication of Pheretima [19–21] has so far aiming at the identification of single crude drug. For example, Chen WM et al. [21] have established a rapid and accurate method to authenticate a genuine species (*A. aspergillus*) in the crude drug of Pheretima using primers at 12S rRNA region.

The present study is the first to use species-specific sites from DNA barcode to identify Pheretima in NXTC, a complex prescription without effective method to control the quality of its animal drugs. In consideration of the DNA degradation during processing and preservation, species-specific primers for Pheretima were designed to target amplicons of small sizes. COI gene was selected for amplification owing to its advantages in providing deeper phylogenetic insights than any other mitochondrial genes [17] and numerous available sequences in GenBank database.

Compared with the identification of single animal drug, one of the difficulties that we encountered with NXTC is the DNA extraction efficiency. Since NXTC contains thirteen plant drugs which account for 83.3% of the components, the large number of plant secondary metabolites are likely to cause difficulty in DNA extraction. Hence, a kit for plant DNA extraction was used to remove the plant secondary metabolites, and proteinase K was then added to digest the animal drugs. Results showed that DNA from the animal drugs was detected with the DNA concentration of NXTC at 50 ng/μl of all the specific primers (Figs. 3 and 4).

Admittedly, more approaches with higher resolution, sensitivity, and throughput can be used for the DNA analysis. For instance, Arulandhu et al. [28] have shown that a multi-locus DNA metabarcoding method based on Illumina MiSeq amplicon sequencing can reproducibly identify plant and animal species in highly complex products such as traditional medicines. Besides, Coghlan et al. [29] used high-throughput sequencing to identify species in 15 complex TCMs presented in the form of powders, tablets, and capsules and revealed that some of them contained currently endangered and protected animals’ DNA. However, methods that applied to the quality control of TCM generally should be rapid, with good universality and easy to operate. And these technologies are relatively costly and expertise-dependent, which may not suitable for bulk detection of Chinese patent medicine. Agarose electrophoresis of the PCR-ed samples is indeed less sensitive than those approaches, but the result of sensitivity test (Figs. 3 and 4) showed that the method we developed is sensitive enough for the identification of Pheretima.

In general, our work has provided an effective and rapid method to authenticate Pheretima in NXTC, which is a progress compared with the current quality control of Pheretima in NXTC by allowing the manufacturer to easily confirm the presence of Pheretima in the product. All testing methods have their own limitations. In this case, our method cannot identify some artificial adulterant samples that do not contain DNA and does not yield information regarding the concentration of active ingredients, which are common problems in many species-specific molecular identification methods for traditional Chinese medicine [30]. However, we have already shown that our method can detect the presence of Pheretima in nine different batches of NXTC of the same manufacturer. Suffice to say, if Pheretima DNA cannot be detected in any one batch of NXTC from the same manufacturer in the future, this result should be alarming, and the manufacturer should refer to the retained starting materials for further authentication and tracking. Certainly, more work need to be done with the quality control of NXTC and a combination of DNA barcoding and other methods like chemical analysis is necessary for a comprehensive quality assessment of Pheretima in NXTC.

## Conclusion

As one of the minister drugs of NXTC, Pheretima is of great importance to the efficacy of the formula. However, effective method for the quality control of animal drugs, especially the authentication of Pheretima, in NXTC is lacking. In this study, species-specific primers for the two genera of Pheretima were designed to identify this animal drug with small-size amplicons at around 230–250 bp, thereby improving the quality control of NXTC. This work has revealed the possibility of using DNA techniques to authenticate animal drug in a complex prescription.

## Supplementary information

**Additional file 1.** GenBank accession numbers of animal species for primer design.

**Additional file 2.** Multiple alignment of the sequences amplified from the crude drugs of Pheretima with specific primers.

**Additional file 3.** DNA sequences of amplicons and its percentage similarity to NCBI DNA sequences.

## Data Availability

The datasets supporting the conclusions of this article are included within the article and its additional files.

## Abbreviations

NXTC: Naoxintong capsule
TCM: traditional Chinese medicine
COI: mitochondrial cytochrome *c* oxidase subunit I
BLAST: Basic Local Alignment Search Tool
CFDA: China Food and Drug Administration
TLC: thin layer chromatography
