# Supplementary material for: Specific DNA identification of Pheretima in the Naoxintong capsule
Source: Chin Med. 2019 Sep 30;14:41. doi: 10.1186/s13020-019-0264-7 (PMC6767644; doi:10.1186/s13020-019-0264-7)
Supplement: Supplementary file 1 — Additional file 1. GenBank accession numbers of animal species for primer design. [file 13020_2019_264_MOESM1_ESM.docx]

**GenBank accession numbers of animal species for primer design**

**Table S1-1 GenBank accession numbers of Metaphire genus for the primer design of Pheretima**

| Animal species | GenBank accession number | Animal species | GenBank accession number |
| --- | --- | --- | --- |
| *Metaphire guillelmi* * | KT429017.1 | *Metaphire paiwanna* | AY962128.1、AY962122.1 |
| *Metaphire vulgaris* * | KJ137279.1 | *Metaphire tschiliensis* | AY962144.1、KP030716.1 |
| *Metaphire anomala* | KU565251.1 | *Metaphire servina* | AB542653.1 |
| *Metaphire sieboldi* | AB607054.1、AB607049.1 | *Metaphire saxicalcis* | KU565292.1 |
| *Metaphire bununa* | AY962142.1 | *Metaphire soulensis* | AB542664.1、AB542662.1 |
| *Metaphire californica* | AY739339.1 | *Metaphire surinensis* | KU565316.1 |
| *Metaphire feijani* | AY960809.1、AY962161.1 | *Metaphire taiwanensis* | AY962157.1、AY960806.1 |
| *Metaphire birmanica* | KU565262.1 | *Metaphire bahli* | KT626580.1 |
| *Metaphire grandipenes* | KU565265.1 | *Metaphire trutina* | AY739338.1、AY962144.1 |
| *Metaphire magna* | KF205982.1、JX315347.1 | *Metaphire vesiculata* | AB542691.1 |
| *Metaphire nanaoensis* | AY962152.1 | *Metaphire yamadai* | AB542694.1 |
| *Metaphire tecta* | KT252966.1 | *Metaphire trangensis* | KU565317.1 |
| *Metaphire khaoluangensis* | KU565275.1 | *Metaphire wuzhimontis* | JQ904537.1 |
| *Metaphire tahanmonta* | AY962116.1 | *Metaphire songkhlaensis* | KU565294.1 |
| *Metaphire glareosa* | AY962180.1、AY960803.1、AY962167.1 | *Metaphire formosae* | AY739326.1、AY739331.1、AY739333.1 |
| *Metaphire agrestis* | AB542605.1、KX400697.1 、AB542597.1 | *Metaphire peguana* | KC404834.1、KC404831.1、KC404832.1 |
| *Metaphire communissima* | AB542625.1、AB542620.1、AB542622.1 | *Metaphire hilgendorfi* | AB425819.1、KX400614.1、AB542639.1 |
| *Metaphire megascolidioides* | AB482107.2、AB536863.1 | *Metaphire yuhsii* | AY739315.1、AY960799.1、AY739309.1 |

* *Metaphire guillelmi* and *Metaphire vulgaris* are the two of four original species of Pheretima, which are recorded in ChP 2015.

**Table A1-2 GenBank accession numbers of Amynthas genus for the primer design of Pheretima**

| Animal species | GenBank accession number | Animal species | GenBank accession number |
| --- | --- | --- | --- |
| *Amynthas aspergillus* * | KJ830749.1、JN187361.1、JQ820335.2、JQ820338.1、DQ224188.1 | *Amynthas amis* | JX290381.1、JX290413.1、JX290408.1、JX290415.1、JX290387.1、JX290396.1、JX290425.1、KU565179.1 |
| *Amynthas penpuensis* | KC897069.1 | *Amynthas phatubensis* | KU565202.1、KU565203.1 |
| *Amynthas borealis* | KU565183.1 | *Amynthas purpuratus* | AB542524.1、AB542521.1 |
| *Amynthas alexandri* | KU565173.1、KU565176.1 | *Amynthas binoculatus* | AY962184.1 |
| *Amynthas comptus* | KU565184.1 | *Amynthas stricosus* | JX315345.1 |
| *Amynthas taiwumontis* | KC897067.1 | *Amynthas triastriatus* | KF179569.1 |
| *Amynthas cucullatus* | KT429012.1 | *Amynthas tappensis* | AB542545.1、AB542551.1 |
| *Amynthas dactilicus* | KF179575.1、AB542473.1 | *Amynthas thakhantho* | KU565243.1、KU565242.1 |
| *Amynthas fuscatus* | AB542480.1、AB542475.1 | *Amynthas tokioensis* | AB542556.1、KY750705.1 |
| *Amynthas fusing* | LC306645.1 | *Amynthas tontong* | KU565245.1、KU565246.1 |
| *Amynthas glabrus* | AB542481.1 | *Amynthas trapezoides* | JX315346.1 |
| *Amynthas incongruus* | KP030694.1 | *Amynthas wuhumontis* | JQ936599.1、JQ936598.1 |
| *Amynthas hupeiensis* | AB542494.1、KF205454.1 | *Amynthas vittatus* | AB542573.1 |
| *Amynthas kinmenensis* | JQ936596.1 | *Amynthas lalashan* | LC306643.1 |
| *Amynthas zhangi* | JX073676.1、KP030720.1 | *Amynthas lioujia* | LC306648.1 |
| *Amynthas daeari* | KF383293.1 | *Amynthas longicaeca* | KU565191.1、KU565195.1 |
| *Amynthas majia* | LC306650.1 | *Amynthas tayalis* | AY962185.1 |
| *Amynthas mediocus* | KF205405.1 | *Amynthas pectiniferus* | KT429018.1 |
| *Amynthas mayshanensis* | DQ224183.1 | *Amynthas phucheefah* | KU565211.1 |
| *Amynthas mekongianus* | KU565196.1 | *Amynthas omeimontis* | KF205480.1 |
| *Amynthas micronarius* | AB542503.1、AB542498.1 | *Amynthas khaohayod* | KU565190.1 |
| *Amynthas moniliatus* | KF179571.1 | *Amynthas jiriensis* | KT783537.1 |
| *Amynthas morrisi* | AB542517.1、EF077580.1 | *Amynthas hainanicus* | JX315409.1 |
| *Amynthas mutabilitas* | JX290402.1、JX290418.1 | *Amynthas exiguus* | KU565189.1 |
| *Amynthas octopapillatus* | JX081510.1 | *Amynthas endophilus* | KF240560.1 |
| *Amynthas papulosus* | KU565200.1 | *Amynthas yunoshimensis* | AB542581.1、AB542583.1 |
| *Amynthas diaoluomontis* | KF205964.1 | *Amynthas yunlongensis* | KF179581.1 |
| *Amynthas polyglandularis* | KC897063.1、DQ224189.1、KC897065.1 | *Amynthas phaselus* | KX400640.1、AB542519.1、KP030707.1 |
| *Amynthas carnosus* | AB542452.1、AB542453.1、KP030699.1 | *Amynthas gracilis* | AB542484.1、KP214564.1、AB542489.1 |
| *Amynthas corticis* | DQ224190.1、AB542455.1、AB542457.1、AB542460.1、AB542461.1 | *Amynthas tungpuensis* | KU232809.1、KU232795.1、KU232805.1、KU232798.1、KU232811.1 |
| *Amynthas hongyehensis* | JX290400.1、JX290399.1、JX290419.1、JX290411.1 | *Amynthas robustus* | DQ224191.1、AB542532.1、AB542529.1、EF077569.1 |
| *Amynthas wulinensis* | DQ224181.1、DQ224177.1、DQ224175.1 | *Amynthas lini* | DQ224166.1、DQ224173.1、DQ224171.1 |
| *Amynthas minimus* | AB542509.1、AB542507.1、AB542510.1 | *Amynthas nangrongensis* | KU565199.1 |

* *Amynthas aspergillus* is the one of four original species of Pheretima, which is recorded in ChP 2015.
