# Supplementary material for: Specific DNA identification of Pheretima in the Naoxintong capsule
Source: Chin Med. 2019 Sep 30;14:41. doi: 10.1186/s13020-019-0264-7 (PMC6767644; doi:10.1186/s13020-019-0264-7)
Supplement: Supplementary file 2 — Additional file 2. Multiple alignment of the sequences amplified from the crude drugs of Pheretima with specific primers. [file 13020_2019_264_MOESM2_ESM.docx]

**Multiple alignment of the sequences amplified from the crude drugs of Pheretima with specific primers.**

**Fig. S2-1-1**

**Fig. S2-1-2**

**Fig. S2-1-3**

**Fig. S2-1-4**

**Fig. S2-1-5**

**Fig. S2-1-(1-5) Multiple alignment of the sequences amplified from the crude drugs of Pheretima with primers MF2R2 and the public sequences of *Metaphire* genus in GenBank nucleotide database.**

*Metaphire vulgaris* and *Metaphire guillelmi* are two of the original animals of Pheretima recorded in ChP 2015. Amplicons of 232 bp were obtained with primers MF2R2 (COI Metaphire F2 and COI Metaphire R2, in blue), and the sequences of them were compared to the public sequences of *Metaphire* genus in GenBank nucleotide database.

**Fig. S2-2-1**

**Fig. S2-2-2**

**Fig. S2-2-3**

**Fig. S2-2-4**

**Fig. S2-2-5**

**Fig. S2-2-(1-5) Multiple alignment of the sequences amplified from the crude drugs of Pheretima with primers AF3R1 and the public sequences of *Amynthas* genus in GenBank nucleotide database.**

*Amynthas aspergillus* is one of the original animals of Pheretima recorded in ChP 2015. Amplicons of 247 bp were obtained with primers AF3R1 (COI AA F3 and COI AA R1, in red), and the sequences of them were compared to the public sequences of *Amynthas* genus in GenBank nucleotide database. However, the adulterant species, *Metaphire californica* could also be amplified using primers AF3R1with the same size fragment, but the sequence of it is quite different from that of the genuine species.
