# Supplementary material for: Specific DNA identification of Pheretima in the Naoxintong capsule
Source: Chin Med. 2019 Sep 30;14:41. doi: 10.1186/s13020-019-0264-7 (PMC6767644; doi:10.1186/s13020-019-0264-7)
Supplement: Supplementary file 3 — Additional file 3. DNA sequences of amplicons and its percentage similarity to NCBI DNA sequences. [file 13020_2019_264_MOESM3_ESM.docx]

**DNA sequences of amplicons and its percentage similarity to NCBI GenBank DNA sequences**

The nine batches of NXTCs obtained from the company were amplified using species-specific primers of the three animal drugs.

| Medicinal material | Primer pair | Size (bp) | Sample code | DNA sequence (5’→3’) | BLAST result | %  similarity |
| --- | --- | --- | --- | --- | --- | --- |
| Pheretima | MF2R2 | 232 | N1 | TTAGTGTCGTCCGCCGCAGTTGAAAAGGGTGCAGGAACAGGGTGAACAGTATACCCCCCACTAGCAAGAAATATTGCCCATGCTGGGCCTTCAGTAGATCTGGCAATCTTTTCACTCCATCTTGCTGGGGCGTCATCAATTTTGGGAGCTATTAATTTCATCACTACAGTAATTAATATGCGGTGATCGGGACTACGGTTAGAACGAATCCCACTATTTGTGTGGGCAGTAG | *Metaphire vulgaris* | 99.14% |
|  |  |  | N2 | TTAGTGTCGTCCGCCGCAGTTGAAAAGGGTGCAGGAACAGGGTGAACAGTATACCCCCCACTAGCAAGAAATATTGCCCATGCTGGGCCTTCAGTAGATCTGGCAATCTTTTCACTCCATCTTGCTGGGGCGTCATCAATTTTGGGAGCTATTAATTTCATCACTACAGTAATTAATATGCGGTGATCGGGACTACGGTTAGAACGAATCCCACTATTTGTGTGGGCAGTAG | *Metaphire vulgaris* | 99.14% |
|  |  |  | N3 | TTAGTGTCGTCCGCCGCAGTTGAAAAGGGTGCAGGAACAGGGTGAACAGTATACCCCCCACTAGCAAGAAATATTGCCCATGCTGGGCCTTCAGTAGATCTAGCAATCTTTTCACTCCATCTTGCTGGGGCGTCATCAATTTTGGGAGCTATTAATTTCATCACTACAGTAATTAATATGCGGTGATCGGGACTACGGTTAGAACGAATCCCACTATTTGTGTGGGCAGTAG | *Metaphire vulgaris* | 99.57% |
|  |  |  | N4 | TTAGTGTCGTCCGCCGCAGTTGAAAAGGGTGCAGGAACAGGGTGAACAGTATACCCCCCACTAGCAAGAAATATTGCCCATGCTGGGCCTTCAGTAGATCTGGCAATCTTTTCACTCCATCTTGCTGGGGCGTCATCAATTTTGGGAGCTATTAATTTCATCACTACAGTAATTAATATGCGGTGATCGGGACTACGGTTAGAACGAATCCCACTATTTGTGTGGGCAGTAG | *Metaphire vulgaris* | 99.14% |
|  |  |  | N5 | TTAGTGTCGTCCGCCGCAGTTGAAAAGGGTGCAGGAACAGGGTGAACAGTATACCCCCCACTAGCAAGAAATATTGCCCATGCTGGGCCTTCAGTAGATCTGGCAATCTTTTCACTCCATCTTGCTGGGGCGTCATCAATTTTGGGAGCTATTAATTTCATCACTACAGTAATTAATATGCGGTGATCGGGACTACGGTTAGAACGAATCCCACTATTTGTGTGGGCAGTAG | *Metaphire vulgaris* | 99.14% |
|  |  |  | N6 | TTAGTGTCGTCCGCCGCAGTTGAAAAGGGTGCAGGAACAGGGTGAACAGTATACCCCCCACTAGCAAGAAATATTGCCCATGCTGGGCCTTCAGTAGATCTGGCAATCTTTTCACTCCATCTTGCTGGGGCGTCATCAATTTTGGGAGCTATTAATTTCATCACTACAGTAATTAATATGCGGTGATCGGGACTACGGTTAGAACGAATCCCACTATTTGTGTGGGCAGTAG | *Metaphire vulgaris* | 99.14% |
|  |  |  | N7 | TTAGTGTCGTCCGCCGCAGTTGAAAAGGGTGCAGGAACAGGGTGAACAGTATACCCCCCACTAGCAAGAAATATTGCCCATGCTGGGCCTTCAGTAGATCTAGCAATCTTTTCACTCCATCTTGCTGGGGCGTCATCAATTTTGGGAGCTATTAATTTCATCACTACAGTAATTAATATGCGGTGATCGGGACTACGGTTAGAACGAATCCCACTATTTGTGTGGGCAGTAG | *Metaphire vulgaris* | 99.57% |
|  |  |  | N8 | TTAGTGTCGTCCGCCGCAGTTGAAAAGGGTGCAGGAACAGGGTGAACAGTATATCCCCCACTAGCAAGAAATATTGCCCATGCTGGGCCTTCAGTAGATCTGGCAATCTTTTCACTCCATCTTGCTGGGGCGTCATCAATTTTGGGAGCTATTAATTTCATCACTACAGTAATTAATATGCGGTGATCGGGACTACGGTTAGAACGAATCCCACTATTTGTGTGGGCAGTAG | *Metaphire vulgaris* | 99.57% |
|  |  |  | N9 | TTAGTGTCGTCCGCCGCAGTTGAAAAGGGTGCAGGAACAGGGTGAACAGTATATCCCCCACTAGCAAGAAATATTGCCCATGCTGGGCCTTCAGTAGATCTGGCAATCTTTTCACTCCATCTTGCTGGGGCGTCATCAATTTTGGGAGCTATTAATTTCATCACTACAGTAATTAATATGCGGTGATCGGGACTACGGTTAGAACGAATCCCACTATTTGTGTGGGCAGTAG | *Metaphire vulgaris* | 99.57% |
